# Supplementary material for: Comparative Analysis of HPA-Axis Dysregulation and Dynamic Molecular Mechanisms in Acute Versus Chronic Social Defeat Stress
Source: Int J Mol Sci. 2025 Jun 24;26(13):6063. doi: 10.3390/ijms26136063 (PMC12250169; doi:10.3390/ijms26136063)
Supplement: Supplementary file 1 [file ijms-26-06063-s001.zip › ijms-3693311-supplementary.pdf]

Supplementary Figures

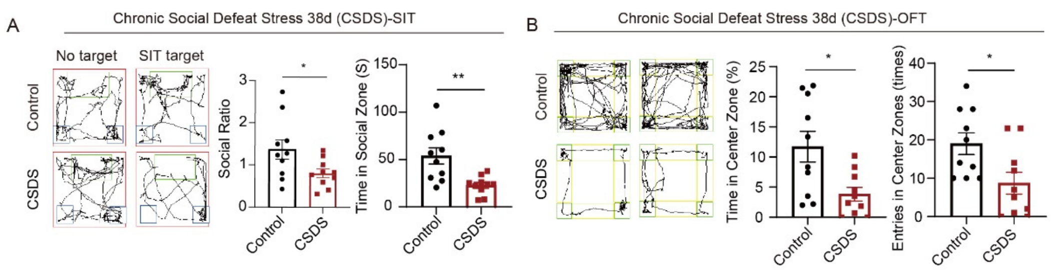

Figure S1. Behavioral assessments of Chronic Social Defeat Stress (CSDS): Social Interaction Test (SIT) and Open filed test (OFT). (A) Social Interaction Test (SIT) path diagrams and statistical analysis of SIT ratios and time spent in the social zone under CSDS (38 days). (Control, n = 10; CSDS, n = 10.) (B) Open filed test (OFT) results showing time spent in the central zone and entries into the central zone under CSDS (38 days). (Control, n = 10; CSDS, n = 10.)

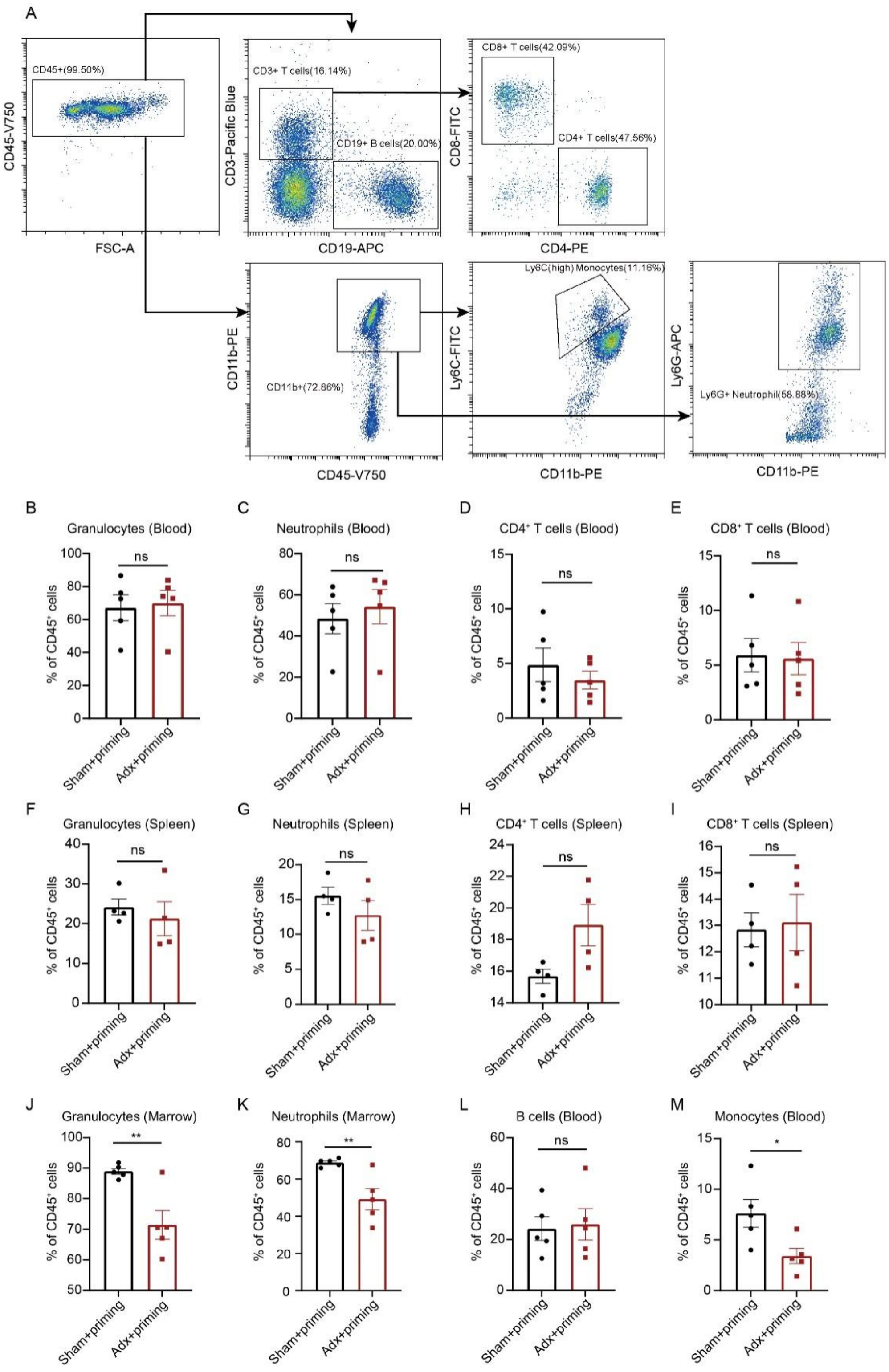

Figure S2. Characterization of Immune Cell Populations in the Spleen and Marrow by Flow Cytometry. (**A**) Gating strategy for blood immune cells. (**B-E**) Statistical analysis of CD11b$^+$ granulocytes, Ly6G$^+$ neutrophils, CD4$^+$ T cells, and CD8$^+$ T in the blood of Sham and ADX mice after priming. (Sham-priming group, n = 5; ADX-priming group, n = 5.) (**F-I**) Statistical analysis of CD11b$^+$ granulocytes, Ly6G$^+$

neutrophils, $CD4^+$ T cells, and $CD8^+$ T cells in the spleen of Sham and ADX mice after priming. (Sham-priming group, n = 4; ADX-priming group, n = 4.) (**J-K**) Statistical analysis of $CD11b^+$ granulocytes and $Ly6G^+$ neutrophils in the bone marrow of Sham and ADX mice after priming. (Sham-priming group, n = 5; ADX-priming group, n = 5.) (**L-M**) Statistical analysis of $CD19^+$ B cells and $Ly6C^+$ monocytes in the blood of Sham and ADX mice after priming. (Sham-priming group, n = 5; ADX-priming group, n = 5.) All data are presented as mean ± SEM and analyzed by two-tailed Student's t test. Significance levels are denoted as follows: ns, not significant; *$p < 0.05$; **$p < 0.01$; ***$p < 0.001$; NS, not significant versus untrained mice.

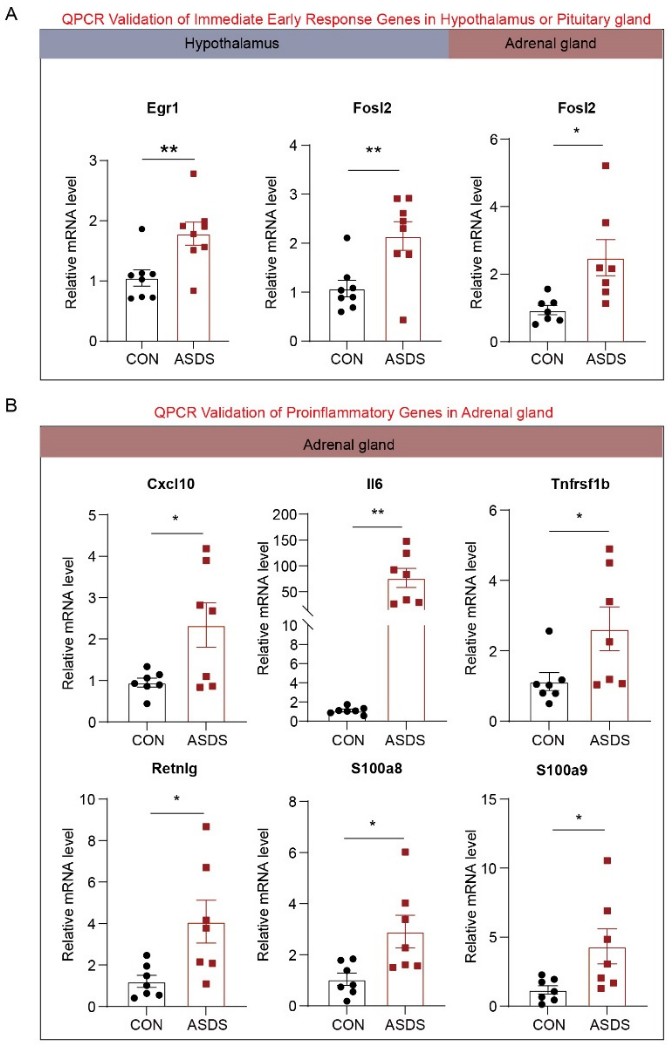

Figure S3. Validation of Gene Expression under Acute Social Defeat Stress (ASDS) using qPCR. (**A**) Validation of immediate early response gene expression in the hypothalamus and adrenal gland under ASDS using qPCR. (**B**) Validation of pro-inflammatory gene expression in the adrenal gland under ASDS using qPCR.

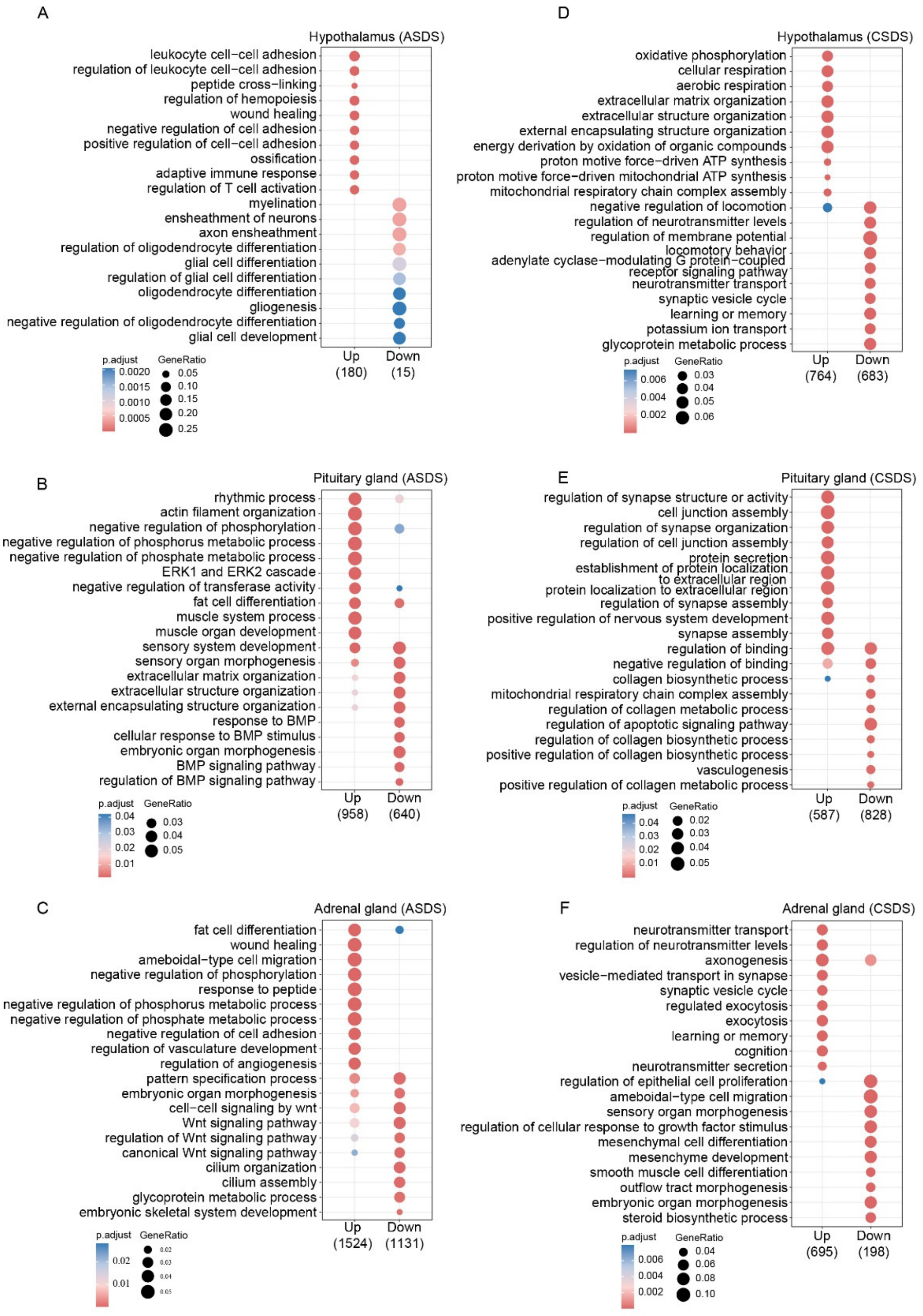

Figure S4. Transcriptomic Alterations in the HPA Axis under Acute or Chronic Social Defeat Stress. (**A-C**) GO enrichment analysis of DEGs in the hypothalamus, pituitary gland, and adrenal gland under ASDS. (**D-F**) GO enrichment analysis of DEGs in the hypothalamus, pituitary gland, and adrenal gland under CSDS.

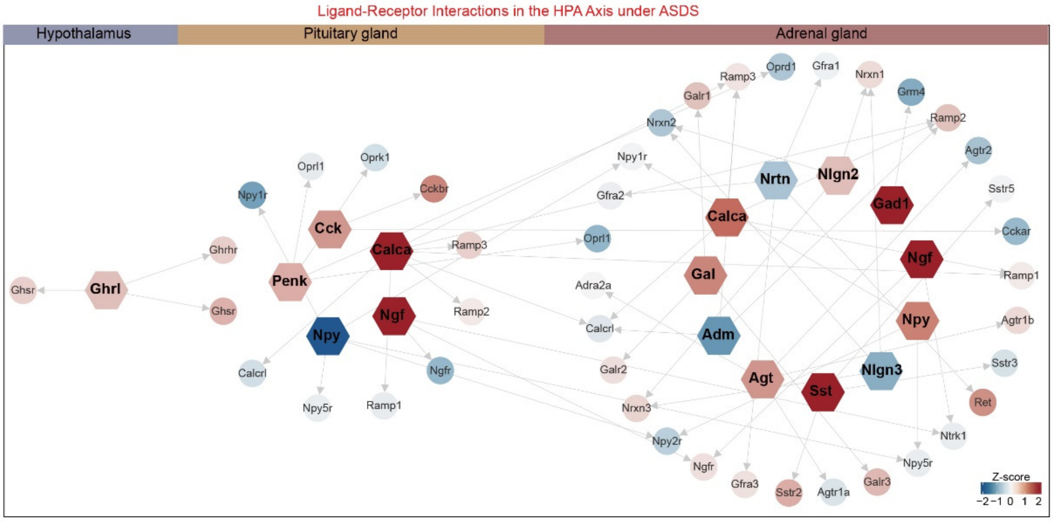

Figure S5. Ligand-Receptor Interactions in the HPA Axis under Acute Social Defeat Stress (ASDS).
